# 2-Hydroxy­ethyl 2-(2,4-dichloro­anilino)-4,4-dimethyl-6-oxocyclo­hex-1-ene­carbo­dithio­ate

**DOI:** 10.1107/S1600536809006199

**Published:** 2009-02-25

**Authors:** El Sayed H. El Ashry, Mohammed R. Amer, M. Raza Shah, Seik Weng Ng

**Affiliations:** a H.E.J. Research Institute of Chemistry, International Center for Chemical and Biological Sciences, University of Karachi, Karachi 75270, Pakistan; b Department of Chemistry, University of Malaya, 50603 Kuala Lumpur, Malaysia

## Abstract

The six-membered cyclo­hexene ring in the title compound, C_17_H_19_Cl_2_NOS_2_, adopts an envelope conformation, with the C atom bearing the two methyl groups representing the flap. This atom deviates by 0.716 (3) Å from the plane passing through the other five atoms of the ring (r.m.s. deviation = 0.072 Å). The mol­ecular conformation is stabilized by an intra­molecular N—H⋯S hydrogen bond. The hydr­oxy group engages in inter­molecular O—H⋯O hydrogen bonding with adjacent acceptor atoms to generate a zigzag chain running along the *c* axis.

## Related literature

For background, see: El Ashry *et al.* (2009 ▶).
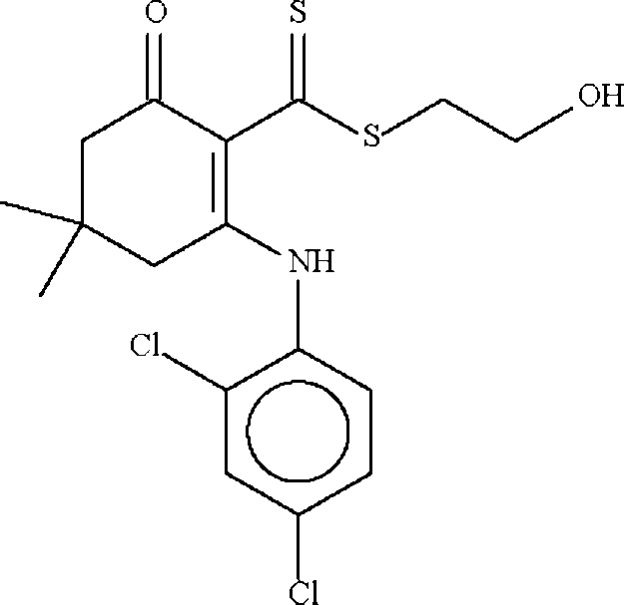


## Experimental

### 

#### Crystal data

C_17_H_19_Cl_2_NO_2_S_2_
*M* *_r_* = 404.35
Monoclinic, 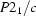

*a* = 11.7310 (2) Å
*b* = 14.0903 (2) Å
*c* = 12.0416 (2) Å
β = 111.245 (1)°
*V* = 1855.13 (5) Å^3^
*Z* = 4
Mo *K*α radiation
μ = 0.59 mm^−1^
*T* = 100 K
0.40 × 0.10 × 0.10 mm

#### Data collection

Bruker SMART APEX diffractometer
Absorption correction: multi-scan (*SADABS*; Sheldrick, 1996 ▶) *T*_min_ = 0.895, *T*_max_ = 0.944
17368 measured reflections
4269 independent reflections
3581 reflections with *I* > 2σ(*I*)
*R*_int_ = 0.031

#### Refinement

*R*[*F* ^2^ > 2σ(*F* ^2^)] = 0.037
*wR*(*F* ^2^) = 0.098
*S* = 1.17
4269 reflections
226 parameters
2 restraints
H atoms treated by a mixture of independent and constrained refinement
Δρ_max_ = 1.48 e Å^−3^
Δρ_min_ = −0.49 e Å^−3^

### 

Data collection: *APEX2* (Bruker, 2008 ▶); cell refinement: *SAINT* (Bruker, 2008 ▶); data reduction: *SAINT*; program(s) used to solve structure: *SHELXS97* (Sheldrick, 2008 ▶); program(s) used to refine structure: *SHELXL97* (Sheldrick, 2008 ▶); molecular graphics: *X-SEED* (Barbour, 2001 ▶); software used to prepare material for publication: *publCIF* (Westrip, 2009 ▶).

## Supplementary Material

Crystal structure: contains datablocks global, I. DOI: 10.1107/S1600536809006199/bt2881sup1.cif

Structure factors: contains datablocks I. DOI: 10.1107/S1600536809006199/bt2881Isup2.hkl

Additional supplementary materials:  crystallographic information; 3D view; checkCIF report

## Figures and Tables

**Table 1:** Hydrogen-bond geometry (Å, °)

*D*—H⋯*A*	*D*—H	H⋯*A*	*D*⋯*A*	*D*—H⋯*A*
O2—H2⋯O1^i^	0.83 (1)	1.92 (1)	2.737 (2)	165 (3)
N1—H1⋯S2	0.88 (1)	2.14 (2)	2.913 (2)	147 (2)

Symmetry code: (i) 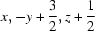.

**Fig. 1.**
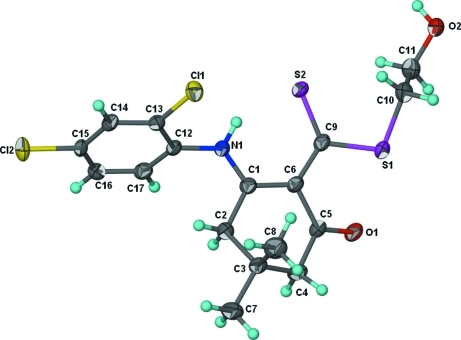
Anisotropic displacement ellipsoid plot (Barbour, 2001) plot of C17H19Cl2NO2S2 at the 70% probability level; hydrogen atoms are drawn as spheres of arbitrary radius.

## supplementary crystallographic information

### Experimental

To a solution of (2,4-dichlorophenylamino)-5,5-dimethyl-cyclohex-2-en-1-one (0.1 mol) in DMSO (20 ml) and sodium hydroxide (0.4 g) in water (1 ml), carbon disulfphide (0.3 mol) was added in the course of 30 minutes. The mixture was stirred for 20 min below 283 K, and then 2-bromoethanol (0.1 mol) was added drop wise at room temperature for 30 min. The reaction mixture was left for 24 h and then diluted with water (200 ml) and acidified with 10% hydrochloric acid. The resulting precipitate was collected by filtration, dried and purified on silica gel column (40% ethyl acetate in hexane) to give yellow crystal (34.5% yield; mp.424 K).

### Refinement

Carbon-bound H-atoms were placed in calculated positions (C—H 0.93 to 0.99 Å) and were included in the refinement in the riding model approximation, with *U*(H) set to 1.2 to 1.5*U*(C).

The amino and hydroxy H-atoms were located in a difference Fourier map, and were refined with distance restraints of N–H 0.88±0.01 and O–H 0.84±0.01 Å; their isotropic displacement parameters were freely refined.

The final difference Fourier map had a large peak at 1 Å from S1, but was otherwise featureless.

### Crystal data

**Table:**

C_17_H_19_Cl_2_NO_2_S_2_	*F*(000) = 840
*M**_r_* = 404.35	*D*_x_ = 1.448 Mg m^−^^3^
Monoclinic, *P*2_1_/*c*	Mo *K*α radiation, λ = 0.71073 Å
Hall symbol: -P 2ybc	Cell parameters from 5593 reflections
*a* = 11.7310 (2) Å	θ = 2.3–28.2°
*b* = 14.0903 (2) Å	µ = 0.59 mm^−^^1^
*c* = 12.0416 (2) Å	*T* = 100 K
β = 111.245 (1)°	Prism, orange
*V* = 1855.13 (5) Å^3^	0.40 × 0.10 × 0.10 mm
*Z* = 4	

### Data collection

**Table:**

Bruker SMART APEX diffractometer	4269 independent reflections
Radiation source: fine-focus sealed tube	3581 reflections with *I* > 2σ(*I*)
graphite	*R*_int_ = 0.031
ω scans	θ_max_ = 27.5°, θ_min_ = 1.9°
Absorption correction: multi-scan (*SADABS*; Sheldrick, 1996)	*h* = −15→15
*T*_min_ = 0.895, *T*_max_ = 0.944	*k* = −18→18
17368 measured reflections	*l* = −15→15

### Refinement

**Table:**

Refinement on *F*^2^	Primary atom site location: structure-invariant direct methods
Least-squares matrix: full	Secondary atom site location: difference Fourier map
*R*[*F*^2^ > 2σ(*F*^2^)] = 0.037	Hydrogen site location: inferred from neighbouring sites
*wR*(*F*^2^) = 0.098	H atoms treated by a mixture of independent and constrained refinement
*S* = 1.17	*w* = 1/[σ^2^(*F*_o_^2^) + (0.0429*P*)^2^ + 0.8915*P*] where *P* = (*F*_o_^2^ + 2*F*_c_^2^)/3
4269 reflections	(Δ/σ)_max_ = 0.001
226 parameters	Δρ_max_ = 1.48 e Å^−^^3^
2 restraints	Δρ_min_ = −0.49 e Å^−^^3^

### Special details

**Table:**

Geometry. All e.s.d.'s (except the e.s.d. in the dihedral angle between two l.s. planes) are estimated using the full covariance matrix. The cell e.s.d.'s are taken into account individually in the estimation of e.s.d.'s in distances, angles and torsion angles; correlations between e.s.d.'s in cell parameters are only used when they are defined by crystal symmetry. An approximate (isotropic) treatment of cell e.s.d.'s is used for estimating e.s.d.'s involving l.s. planes.

### Fractional atomic coordinates and isotropic or equivalent isotropic displacement parameters (Å^2^)

**Table:**

	*x*	*y*	*z*	*U*_iso_*/*U*_eq_	
Cl1	0.50297 (4)	0.66542 (3)	0.81611 (4)	0.02438 (12)	
Cl2	0.69365 (5)	0.33055 (4)	1.00864 (5)	0.02908 (13)	
S1	−0.02899 (5)	0.74647 (4)	0.42522 (4)	0.02988 (14)	
S2	0.11953 (5)	0.71791 (4)	0.68063 (4)	0.02937 (14)	
O1	−0.05234 (12)	0.57916 (11)	0.32339 (12)	0.0255 (3)	
O2	−0.14964 (14)	0.95304 (11)	0.58309 (13)	0.0302 (4)	
H2	−0.123 (2)	0.954 (2)	0.6574 (9)	0.048 (8)*	
N1	0.27644 (14)	0.55532 (12)	0.69084 (14)	0.0203 (3)	
H1	0.250 (2)	0.6051 (12)	0.718 (2)	0.038 (7)*	
C1	0.21902 (17)	0.53820 (13)	0.57550 (17)	0.0188 (4)	
C2	0.26758 (19)	0.45827 (15)	0.52239 (17)	0.0239 (4)	
H2A	0.2247	0.3989	0.5278	0.029*	
H2B	0.3556	0.4495	0.5698	0.029*	
C3	0.25180 (18)	0.47536 (15)	0.39220 (17)	0.0239 (4)	
C4	0.11563 (19)	0.48819 (16)	0.32527 (18)	0.0266 (4)	
H4A	0.1028	0.5110	0.2439	0.032*	
H4B	0.0754	0.4255	0.3176	0.032*	
C5	0.05389 (17)	0.55607 (14)	0.38206 (16)	0.0203 (4)	
C6	0.11713 (16)	0.59172 (14)	0.50288 (16)	0.0191 (4)	
C7	0.2972 (2)	0.38762 (17)	0.3452 (2)	0.0343 (5)	
H7A	0.2836	0.3965	0.2606	0.051*	
H7B	0.2523	0.3315	0.3549	0.051*	
H7C	0.3848	0.3788	0.3901	0.051*	
C8	0.3236 (2)	0.56243 (17)	0.3788 (2)	0.0326 (5)	
H8A	0.3078	0.5734	0.2942	0.049*	
H8B	0.4112	0.5516	0.4212	0.049*	
H8C	0.2977	0.6181	0.4124	0.049*	
C9	0.07364 (17)	0.67738 (14)	0.54093 (16)	0.0203 (4)	
C10	−0.0347 (2)	0.85810 (15)	0.49607 (19)	0.0289 (5)	
H10A	0.0452	0.8699	0.5604	0.035*	
H10B	−0.0496	0.9099	0.4370	0.035*	
C11	−0.13386 (19)	0.85877 (16)	0.5476 (2)	0.0293 (5)	
H11A	−0.2114	0.8358	0.4873	0.035*	
H11B	−0.1115	0.8158	0.6173	0.035*	
C12	0.37500 (16)	0.50105 (14)	0.76960 (16)	0.0182 (4)	
C13	0.48643 (17)	0.54444 (13)	0.83201 (16)	0.0175 (4)	
C14	0.58495 (16)	0.49276 (14)	0.90679 (16)	0.0183 (4)	
H14	0.6609	0.5228	0.9489	0.022*	
C15	0.56972 (17)	0.39647 (14)	0.91836 (16)	0.0189 (4)	
C16	0.45843 (18)	0.35196 (14)	0.86133 (17)	0.0216 (4)	
H16	0.4491	0.2861	0.8727	0.026*	
C17	0.36107 (17)	0.40498 (14)	0.78745 (17)	0.0217 (4)	
H17	0.2841	0.3754	0.7486	0.026*	

### Atomic displacement parameters (Å^2^)

**Table:**

	*U*^11^	*U*^22^	*U*^33^	*U*^12^	*U*^13^	*U*^23^
Cl1	0.0248 (2)	0.0197 (2)	0.0274 (3)	0.00018 (18)	0.00800 (19)	0.00396 (18)
Cl2	0.0231 (2)	0.0295 (3)	0.0298 (3)	0.0102 (2)	0.0038 (2)	0.0088 (2)
S1	0.0367 (3)	0.0309 (3)	0.0171 (2)	0.0127 (2)	0.0039 (2)	0.0018 (2)
S2	0.0306 (3)	0.0345 (3)	0.0157 (2)	0.0153 (2)	−0.0004 (2)	−0.0050 (2)
O1	0.0188 (7)	0.0343 (8)	0.0179 (7)	0.0032 (6)	0.0001 (5)	−0.0036 (6)
O2	0.0316 (8)	0.0319 (8)	0.0202 (8)	0.0137 (7)	0.0011 (6)	−0.0012 (6)
N1	0.0167 (8)	0.0233 (9)	0.0166 (8)	0.0055 (7)	0.0009 (6)	−0.0029 (6)
C1	0.0163 (8)	0.0212 (9)	0.0179 (9)	0.0001 (7)	0.0051 (7)	−0.0012 (7)
C2	0.0247 (10)	0.0254 (10)	0.0199 (10)	0.0071 (8)	0.0062 (8)	−0.0008 (8)
C3	0.0234 (10)	0.0294 (11)	0.0178 (9)	0.0062 (8)	0.0063 (8)	−0.0033 (8)
C4	0.0253 (10)	0.0339 (11)	0.0178 (9)	0.0047 (9)	0.0045 (8)	−0.0074 (8)
C5	0.0199 (9)	0.0237 (10)	0.0152 (9)	−0.0006 (8)	0.0037 (7)	0.0002 (7)
C6	0.0165 (9)	0.0247 (10)	0.0142 (9)	0.0017 (7)	0.0031 (7)	−0.0015 (7)
C7	0.0358 (12)	0.0372 (13)	0.0290 (11)	0.0103 (10)	0.0107 (10)	−0.0094 (10)
C8	0.0306 (11)	0.0395 (13)	0.0313 (12)	0.0024 (10)	0.0155 (9)	−0.0023 (10)
C9	0.0154 (9)	0.0271 (10)	0.0160 (9)	0.0029 (7)	0.0027 (7)	−0.0006 (7)
C10	0.0365 (12)	0.0255 (10)	0.0234 (10)	0.0071 (9)	0.0093 (9)	0.0036 (8)
C11	0.0247 (10)	0.0307 (11)	0.0279 (11)	0.0055 (9)	0.0038 (9)	0.0000 (9)
C12	0.0157 (9)	0.0236 (9)	0.0137 (8)	0.0038 (7)	0.0033 (7)	−0.0014 (7)
C13	0.0192 (9)	0.0192 (9)	0.0152 (8)	0.0006 (7)	0.0075 (7)	0.0003 (7)
C14	0.0136 (8)	0.0248 (10)	0.0156 (9)	−0.0002 (7)	0.0043 (7)	−0.0001 (7)
C15	0.0182 (9)	0.0233 (9)	0.0144 (9)	0.0071 (7)	0.0048 (7)	0.0033 (7)
C16	0.0246 (10)	0.0188 (9)	0.0212 (10)	0.0015 (8)	0.0081 (8)	−0.0010 (7)
C17	0.0184 (9)	0.0245 (10)	0.0198 (9)	−0.0018 (8)	0.0042 (7)	−0.0039 (8)

### Geometric parameters (Å, °)

**Table:**

Cl1—C13	1.7341 (19)	C5—C6	1.462 (3)
Cl2—C15	1.7358 (18)	C6—C9	1.448 (3)
S1—C9	1.7671 (19)	C7—H7A	0.9800
S1—C10	1.802 (2)	C7—H7B	0.9800
S2—C9	1.6701 (19)	C7—H7C	0.9800
O1—C5	1.233 (2)	C8—H8A	0.9800
O2—C11	1.428 (3)	C8—H8B	0.9800
O2—H2	0.834 (10)	C8—H8C	0.9800
N1—C1	1.328 (2)	C10—C11	1.504 (3)
N1—C12	1.424 (2)	C10—H10A	0.9900
N1—H1	0.879 (10)	C10—H10B	0.9900
C1—C6	1.416 (3)	C11—H11A	0.9900
C1—C2	1.505 (3)	C11—H11B	0.9900
C2—C3	1.530 (3)	C12—C17	1.389 (3)
C2—H2A	0.9900	C12—C13	1.391 (3)
C2—H2B	0.9900	C13—C14	1.387 (3)
C3—C4	1.517 (3)	C14—C15	1.382 (3)
C3—C8	1.529 (3)	C14—H14	0.9500
C3—C7	1.533 (3)	C15—C16	1.385 (3)
C4—C5	1.505 (3)	C16—C17	1.385 (3)
C4—H4A	0.9900	C16—H16	0.9500
C4—H4B	0.9900	C17—H17	0.9500
			
C9—S1—C10	103.83 (10)	C3—C8—H8B	109.5
C11—O2—H2	106.8 (19)	H8A—C8—H8B	109.5
C1—N1—C12	125.53 (16)	C3—C8—H8C	109.5
C1—N1—H1	115.2 (17)	H8A—C8—H8C	109.5
C12—N1—H1	119.3 (17)	H8B—C8—H8C	109.5
N1—C1—C6	122.96 (17)	C6—C9—S2	125.58 (14)
N1—C1—C2	116.98 (16)	C6—C9—S1	115.29 (13)
C6—C1—C2	120.05 (16)	S2—C9—S1	118.99 (11)
C1—C2—C3	113.19 (16)	C11—C10—S1	111.57 (16)
C1—C2—H2A	108.9	C11—C10—H10A	109.3
C3—C2—H2A	108.9	S1—C10—H10A	109.3
C1—C2—H2B	108.9	C11—C10—H10B	109.3
C3—C2—H2B	108.9	S1—C10—H10B	109.3
H2A—C2—H2B	107.8	H10A—C10—H10B	108.0
C4—C3—C8	111.24 (18)	O2—C11—C10	109.44 (18)
C4—C3—C2	106.04 (16)	O2—C11—H11A	109.8
C8—C3—C2	111.58 (17)	C10—C11—H11A	109.8
C4—C3—C7	109.87 (17)	O2—C11—H11B	109.8
C8—C3—C7	109.25 (18)	C10—C11—H11B	109.8
C2—C3—C7	108.80 (17)	H11A—C11—H11B	108.2
C5—C4—C3	114.95 (16)	C17—C12—C13	118.99 (17)
C5—C4—H4A	108.5	C17—C12—N1	120.92 (17)
C3—C4—H4A	108.5	C13—C12—N1	120.07 (17)
C5—C4—H4B	108.5	C14—C13—C12	121.26 (17)
C3—C4—H4B	108.5	C14—C13—Cl1	119.19 (14)
H4A—C4—H4B	107.5	C12—C13—Cl1	119.55 (14)
O1—C5—C6	121.51 (17)	C15—C14—C13	118.24 (17)
O1—C5—C4	117.40 (17)	C15—C14—H14	120.9
C6—C5—C4	121.08 (16)	C13—C14—H14	120.9
C1—C6—C9	124.33 (17)	C14—C15—C16	121.83 (17)
C1—C6—C5	116.24 (17)	C14—C15—Cl2	118.61 (14)
C9—C6—C5	119.43 (16)	C16—C15—Cl2	119.55 (15)
C3—C7—H7A	109.5	C15—C16—C17	118.94 (18)
C3—C7—H7B	109.5	C15—C16—H16	120.5
H7A—C7—H7B	109.5	C17—C16—H16	120.5
C3—C7—H7C	109.5	C16—C17—C12	120.60 (18)
H7A—C7—H7C	109.5	C16—C17—H17	119.7
H7B—C7—H7C	109.5	C12—C17—H17	119.7
C3—C8—H8A	109.5		
			
C12—N1—C1—C6	176.51 (18)	C1—C6—C9—S1	164.93 (16)
C12—N1—C1—C2	−4.0 (3)	C5—C6—C9—S1	−15.1 (2)
N1—C1—C2—C3	−147.64 (18)	C10—S1—C9—C6	−166.31 (15)
C6—C1—C2—C3	31.9 (3)	C10—S1—C9—S2	9.62 (15)
C1—C2—C3—C4	−58.6 (2)	C9—S1—C10—C11	−89.89 (16)
C1—C2—C3—C8	62.7 (2)	S1—C10—C11—O2	−169.55 (13)
C1—C2—C3—C7	−176.75 (17)	C1—N1—C12—C17	−59.2 (3)
C8—C3—C4—C5	−73.5 (2)	C1—N1—C12—C13	122.2 (2)
C2—C3—C4—C5	48.0 (2)	C17—C12—C13—C14	3.2 (3)
C7—C3—C4—C5	165.45 (19)	N1—C12—C13—C14	−178.21 (17)
C3—C4—C5—O1	170.11 (18)	C17—C12—C13—Cl1	−176.94 (14)
C3—C4—C5—C6	−10.9 (3)	N1—C12—C13—Cl1	1.7 (2)
N1—C1—C6—C9	8.1 (3)	C12—C13—C14—C15	−0.2 (3)
C2—C1—C6—C9	−171.37 (18)	Cl1—C13—C14—C15	179.86 (14)
N1—C1—C6—C5	−171.89 (18)	C13—C14—C15—C16	−2.5 (3)
C2—C1—C6—C5	8.6 (3)	C13—C14—C15—Cl2	178.31 (14)
O1—C5—C6—C1	159.28 (18)	C14—C15—C16—C17	2.3 (3)
C4—C5—C6—C1	−19.6 (3)	Cl2—C15—C16—C17	−178.56 (15)
O1—C5—C6—C9	−20.7 (3)	C15—C16—C17—C12	0.7 (3)
C4—C5—C6—C9	160.35 (18)	C13—C12—C17—C16	−3.4 (3)
C1—C6—C9—S2	−10.7 (3)	N1—C12—C17—C16	177.98 (17)
C5—C6—C9—S2	169.31 (15)		

### Hydrogen-bond geometry (Å, °)

**Table:**

*D*—H···*A*	*D*—H	H···*A*	*D*···*A*	*D*—H···*A*
O2—H2···O1^i^	0.83 (1)	1.92 (1)	2.737 (2)	165 (3)
N1—H1···S2	0.88 (1)	2.14 (2)	2.913 (2)	147 (2)

Symmetry codes: (i) *x*, −*y*+3/2, *z*+1/2.
